# Does function trump bioinformatics in Brugada syndrome-associated *SCN5A* mutation calling? Patients, computers, and patches

**DOI:** 10.1093/eurheartj/ehab292

**Published:** 2021-08-01

**Authors:** Arthur A M Wilde, Cheng-I Wu

**Affiliations:** Amsterdam UMC, University of Amsterdam, Heart Center, Department of Clinical and Experimental Cardiology, Amsterdam Cardiovascular Sciences, Amsterdam, The Netherlands; Amsterdam UMC, University of Amsterdam, Heart Center, Department of Clinical and Experimental Cardiology, Amsterdam Cardiovascular Sciences, Amsterdam, The Netherlands; Heart Rhythm Center, Division of Cardiology, Department of Medicine, Taipei Veterans General Hospital, Taipei, Taiwan

**This editorial refers to ‘Functionally validated *SCN5A* variants allow interpretation of pathogenicity and prediction of lethal events in Brugada syndrome’, by T. Ishikawa *et al*., doi:10.1093/eurheartj/ehab254.**

**Graphical Abstract:**
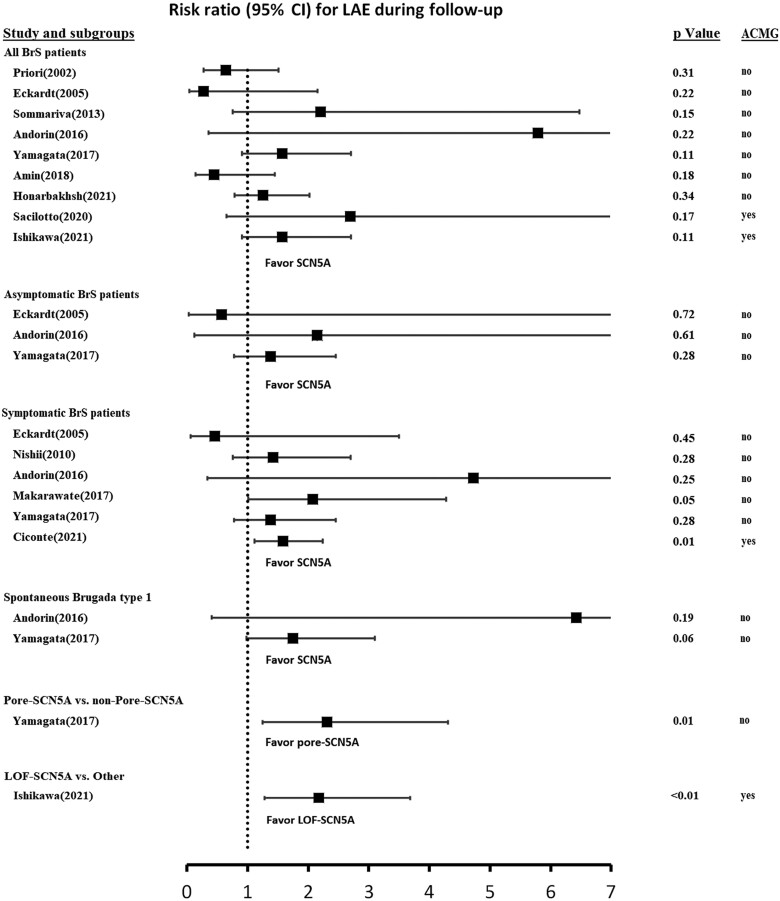
The association of *SCN5A* mutation and life-threatening arrhythmic events (LAEs) was assessed in different studies and subgroups, and relative risk was compared in the forest plot. ACMG refers to the American College of Medical Genetics and Genomics criteria. CI, confidence interval; LOF, loss of function.

*SCN5A*, the gene encoding the α-subunit of the cardiac sodium channel, plays a key role in Brugada syndrome (BrS). Although the associated pathophysiological pathways are incompletely understood, a causal role for pathogenic loss-of-function (LOF) *SCN5A* variants, identified in ∼20–30% of BrS patients, is widely accepted to play a role in this intriguing entity.^1^ BrS patients are at risk for life-threatening arrhythmic events (LAEs), but defining the precise individual risk profile has proven to be very difficult.^2^ The contribution of *SCN5A* rare variant carriership to the risk of LAEs is controversial.^2^ However, a recent systematic review provided evidence for a potential role in determination of risk in specific risk subgroups.^3^ For example, a positive correlation was observed in the Asian population (but not in the Caucasian population), in symptomatic patients and not in asymptomatic patients, and in patients with a spontaneous type 1 pattern.^3^ Unfortunately, with the exception of the ethnicity data, the comparisons were based on <300 patients with in total no more than 75 patients with an *SCN5A* variant.^3^ It can thus safely be concluded that overall, at present, it is not clear whether the presence of a pathogenic *SCN5A* variant contributes to actual risk.

A potential disturbing factor in establishing a role for *SCN5A* variant status in risk for cardiac events is the assignment of pathogenicity for the identified variant. *SCN5A* is a gene with a relatively high background rate of rare amino acid-altering genetic variants (∼2% for whites and ∼5% for non-whites)^4^ and, while proper assignment of causation for *SCN5A* variants is important, it represents a major challenge, particularly for missense variants. Importantly, including seemingly pathogenic, but effectively innocent, variants in studies investigating the relationship with cardiac events may dilute the actual contribution of *SCN5A* variant status. Up till a few years ago, bioinformatic tools were predominantly used to aid variant adjudication.^5^ These have now been replaced by variant classification schemes based on the American College of Medical Genetics and Genomics (ACMG) guidelines, although many of the identified variants end up being classified as a variant of unknown significance (VUS). More recently, high-throughput functional readouts have become available to aid variant classification. Indeed, by high-throughput automated patch–clamp analysis on 73 *SCN5A* variants, 63 of them identified in BrS patients, 35 of 61 variants assigned a VUS status could be upgraded to a likely pathogenic status whereas 14 could be downgraded to benign/likely benign status.^6^ These results presumably reflect the considerable weight of functional evidence in the variant adjudication process according to the ACMG guidelines. Because of unavailability of clinical data in such studies, it is unknown whether patients with upgraded variants were more seriously affected compared with patients with downgraded variants.

This important piece of information is presented in a study by Ishikawa and co-workers in this issue of the *European Heart Journal*.^7^ Sixty Japanese BrS patients with 55 unique *SCN5A* variants were studied. A literature survey identified a functional LOF effect of 33 of these variants. The remaining 22 variants, which were classified as VUS, were subject to functional studies (by old fashioned manual methods), uncovering normal channel activity for almost half. Of note, among the latter were variants that would have been classified as pathogenic in the days when assignment of pathogenicity rested on outcome of bioinformatic analysis and/or on their low frequency or absence in the general population, i.e. the pre-ACMG era. Subsequently, and not surprisingly, the authors demonstrated (figure 4B in their manuscript) that the 45 patients with 40 different LOF variants had more LAEs upon follow-up compared with the 15 patients with innocent *SCN5A* variants.

While one needs to acknowledge that heterologous expression studies do not always unmask the mechanism of aberrant sodium channels,^8^ the importance of the study lies in the observation that in the absence of functional data, a potential 25% of ‘*SCN5A*-associated’ BrS patients (15 out of 60) would have been misclassified as causal and, which, as argued above, may lead to an underestimation of the effect of *SCN5A* status on the risk of LAEs (compare their figure 4A and C). It is likely that this explains why, in many earlier studies, the association of *SCN5A* variant status with phenotype severity did not reach statistical significance (*Graphical Abstract*), with the exception of only three studies (out of 12)^7^ ^,^ ^9^ ^,^ ^10^ (see Supplementary material at *European Heart Journal* online on quantitative data and the references of all these studies). In two of these,^7^ ^,^ ^9^ including the present study,^7^ function (either measured or assumed on safe criteria) was included as a determinant of pathogenicity, and in the third one variant calling was based on the ACMG criteria.^10^ The third study is also of importance because it indicated that *SCN5A* status is the strongest predictor of the epicardial area size, which is the substrate of the associated arrhythmias.^10^

The second part of the study involves whole-exome screening for additional rare variants in an additional cohort of BrS patients (288 patients), with the aim of identifying variants that play an eventual role in the risk for non-*SCN5A* BrS patients. No genes enriched with variants in the BrS cohort were identified.^7^ As to risk of LAEs, the focus was on rare variants in the ‘Brugada-susceptibility genes’, i.e. the 22 genes that were recently discarded as such.^2^ Rare variants were identified in 17 of these 22 genes, but the number of rare variants was comparable with that in the control sample, and BrS patients with these rare variants had a similar prognosis compared with BrS patients without.^7^

In conclusion, this study provides strong evidence that a pathogenic *SCN5A* variant contributes to the severity of the phenotype but that functional studies are needed to call *SCN5A* variants. It seems likely that in earlier studies, including our own, where the presence of a ‘pathogenic *SCN5A* variant’ did not contribute to risk, a substantial subset of *SCN5A* variants were not correctly called and therefore diluted the prognostic effect.

## Supplementary material

Supplementary material is available at *European Heart Journal* online.

## Supplementary Material

ehab292_Supplementary_Materials

## Notes

The opinions expressed in this article are not necessarily those of the Editors of the *European Heart Journal* or of the European Society of Cardiology.
