# Supplementary material for: Does function trump bioinformatics in Brugada syndrome-associated SCN5A mutation calling? Patients, computers, and patches
Source: Eur Heart J. 2021 Aug 1;42(29):2864–5. doi: 10.1093/eurheartj/ehab292 (PMC8325777; doi:10.1093/eurheartj/ehab292)
Supplement: ehab292_Supplementary_Materials [file ehab292_supplementary_materials.docx]

**Supplemental materials**

| Supplemental Table 1. The association between mutation status and life-threatening arrhythmic events in different subgroups | | | | | | |
| --- | --- | --- | --- | --- | --- | --- |
|  | *SCN5A*(+) | | *SCN5A*(-) | |  |  |
| Subgroups and studies (year) | Patients with LAE-N | Total patients-N | Patients with LAE-N | Total patients-N | Relative risk (95% CI) | P-value |
| All BrS patients | | | | | | |
| Priori (2002)^1^ | 7 | 84 | 15 | 116 | 0.64(0.27-1.51) | 0.31 |
| Eckardt (2005)^2^ | 1 | 57 | 8 | 126 | 0.28(0.04-2.16) | 0.22 |
| Sommariva (2013)^3^ | 4 | 17 | 8 | 75 | 2.21(0.75-6.49) | 0.15 |
| Andorin (2016)^4^ | 9 | 58 | 0 | 17 | 5.80(0.35-94.80) | 0.22 |
| Yamagata (2017)^5^ | 13 | 60 | 49 | 355 | 1.57(0.91-2.71) | 0.11 |
| Amin (2018)^6^ | 3 | 88 | 25 | 328 | 0.45(0.14-1.45) | 0.18 |
| Honarbakhsh (2021)^7^ | 21 | 175 | 53 | 556 | 1.26(0.78-2.03) | 0.34 |
| Sacilotto (2020)^8^ | 3 | 10 | 3 | 27 | 2.70(0.65-11.24) | 0.17 |
| Ishikawa (2021) | 13 | 60 | 49 | 355 | 1.57(0.91-2.71) | 0.11 |
| Asymptomatic BrS patients | | | | | | |
| Eckardt (2005)^2^ | 0 | 38 | 1 | 65 | 0.56(0.02-13.51) | 0.72 |
| Andorin (2016)^4^ | 3 | 48 | 0 | 14 | 2.14(0.12-39.19) | 0.61 |
| Yamagata (2017)^5^ | 10 | 30 | 38 | 157 | 1.38(0.77-2.45) | 0.28 |
| Symptomatic BrS patients | | | | | | |
| Eckard t(2005)^2^ | 1 | 19 | 7 | 61 | 0.46(0.06-3.50) | 0.45 |
| Nishii (2010)^9^ | 5 | 8 | 18 | 41 | 1.42(0.75-2.70) | 0.28 |
| Andorin (2016)^4^ | 6 | 10 | 0 | 3 | 4.73(0.34-66.18) | 0.25 |
| Makarawate (2017)^9^ | 8 | 13 | 8 | 27 | 2.08(1.01-4.28) | 0.05 |
| Yamagata (2017)^5^ | 10 | 30 | 38 | 157 | 1.38(0.77-2.45) | 0.28 |
| Ciconte (2021)^10^ | 26 | 49 | 49 | 146 | 1.58(1.12-2.24) | 0.01 |
| Spontaneous Brugada type 1 | | | | | |  |
| Andorin (2016)^4^ | 7 | 20 | 0 | 8 | 6.42(0.41-100.98) | 0.19 |
| Yamagata (2017)^5^ | 12 | 48 | 36 | 251 | 1.74(0.98-3.10) | 0.06 |
| Pore-*SCN5A* | Pore-*SCN5A*(+) | | Pore-*SCN5A*(-) | |  |  |
| Yamagata (2017)^5^ | 8 | 25 | 54 | 390 | 2.31(1.24-4.31) | 0.01 |
|  | LOF-SCN5A(+) | | Non-LOF-SCN5A(+) and SCN5A(-) | |  |  |
| Ishikawa (2021) | 13 | 45 | 49 | 370 | 2.18(1.29-3.70) | <0.01 |
| BrS, Brugada syndrome; CI, confidence interval; LAE, life-threatening arrhythmic event; LOF, loss of function. | | | | | | |

Supplemental References

1. Priori SG, Napolitano C, Gasparini M, Pappone C, Della Bella P, Giordano U, Bloise R, Giustetto C, De Nardis R, Grillo M, Ronchetti E, Faggiano G, Nastoli J. Natural history of Brugada syndrome: insights for risk stratification and management. Circulation 2002; **105**: 1342-1347.

2. Eckardt L, Probst V, Smits JP, Bahr ES, Wolpert C, Schimpf R, Wichter T, Boisseau P, Heinecke A, Breithardt G, Borggrefe M, LeMarec H, Böcker D, Wilde AA. Long-term prognosis of individuals with right precordial ST-segment-elevation Brugada syndrome. Circulation 2005; **111**: 257-263.

3. Sommariva E, Pappone C, Martinelli Boneschi F, Di Resta C, Rosaria Carbone M, Salvi E, Vergara P, Sala S, Cusi D, Ferrari M, Benedetti S. Genetics can contribute to the prognosis of Brugada syndrome: a pilot model for risk stratification. Eur J Hum Genet. 2013; **21**: 911-917.

4. Andorin A, Behr ER, Denjoy I, Crotti L, Dagradi F, Jesel L, Sacher F, Petit B, Mabo P, Maltret A, Wong LC, Degand B, Bertaux G, Maury P, Dulac Y, Delasalle B, Gourraud JB, Babuty D, Blom NA, Schwartz PJ, Wilde AA, Probst V. Impact of clinical and genetic findings on the management of young patients with Brugada syndrome. Heart Rhythm 2016; **13**: 1274-1282.

5. Yamagata K, Horie M, Aiba T, Ogawa S, Aizawa Y, Ohe T, Yamagishi M, Makita N, Sakurada H, Tanaka T, Shimizu A, Hagiwara N, Kishi R, Nakano Y, Takagi M, Makiyama T, Ohno S, Fukuda K, Watanabe H, Morita H, Hayashi K, Kusano K, Kamakura S, Yasuda S, Ogawa H, Miyamoto Y, Kapplinger JD, Ackerman MJ, Shimizu W. Genotype-Phenotype Correlation of SCN5A Mutation for the Clinical and Electrocardiographic Characteristics of Probands With Brugada Syndrome: A Japanese Multicenter Registry. Circulation 2017; **135**: 2255-2270.

6. Amin AS, Reckman YJ, Arbelo E, Spanjaart AM, Postema PG, Tadros R, Tanck MW, Van den Berg MP, Wilde AAM, Tan HL. SCN5A mutation type and topology are associated with the risk of ventricular arrhythmia by sodium channel blockers. Int J Cardiol 2018; **266**: 128-132.

7. Honarbakhsh S, Providencia R, Garcia-Hernandez J, Martin CA, Hunter RJ, Lim WY, Kirkby C, Graham AJ, Sharifzadehgan A, Waldmann V, Marijon E, Munoz-Esparza C, Lacunza J, Gimeno-Blanes JR, Ankou B, Chevalier P, Antonio N, Elvas L, Castelletti S, Crotti L, Schwartz P, Scanavacca M, Darrieux F, Sacilotto L, Mueller-Leisse J, Veltmann C, Vicentini A, Demarchi A, Cortez-Dias N, Antonio PS, de Sousa J, Adragao P, Cavaco D, Costa FM, Khoueiry Z, Boveda S, Sousa MJ, Jebberi Z, Heck P, Mehta S, Conte G, Ozkartal T, Auricchio A, Lowe MD, Schilling RJ, Prieto-Merino D, Lambiase PD. A Primary Prevention Clinical Risk Score Model for Patients With Brugada Syndrome (BRUGADA-RISK). JACC Clin Electrophysiol 2021; **7**: 210-222.

8. Sacilotto L, Scanavacca MI, Olivetti N, Lemes C, Pessente GD, Wulkan F, Hachul DT, Krieger JE, Pereira AC, Darrieux FCC. Low rate of life-threatening events and limitations in predicting invasive and noninvasive markers of symptoms in a cohort of type 1 Brugada syndrome patients: Data and insights from the GenBra registry. J Cardiovasc Electrophysiol 2020; **31**: 2920-2928.

9. Nishii N, Ogawa M, Morita H, Nakamura K, Banba K, Miura D, Kumagai N, Matsunaga A, Kawamura H, Urakawa S, Miyaji K, Nagai M, Satoh K, Nakagawa K, Tanaka M, Hiramatsu S, Tada T, Murakami M, Nagase S, Kohno K, Kusano KF, Saku K, Ohe T, Ito H. SCN5A mutation is associated with early and frequent recurrence of ventricular fibrillation in patients with Brugada syndrome. Circ J 2010; **74**: 2572-2578.

10. Ciconte G, Monasky MM, Santinelli V, Micaglio E, Vicedomini G, Anastasia L, Negro G, Borrelli V, Giannelli L, Santini F, de Innocentiis C, Rondine R, Locati ET, Bernardini A, Mazza BC, Mecarocci V, Ćalović Ž, Ghiroldi A, D'Imperio S, Benedetti S, Di Resta C, Rivolta I, Casari G, Petretto E, Pappone C. Brugada syndrome genetics is associated with phenotype severity. Eur Heart J 2021; **42**: 1082-1090.
